# Supplementary material for: Positioning of nucleosomes containing γ-H2AX precedes active DNA demethylation and transcription initiation
Source: Nat Commun. 2021 Feb 16;12:1072. doi: 10.1038/s41467-021-21227-y (PMC7886895; doi:10.1038/s41467-021-21227-y)
Supplement: Supplementary file 1 — Supplementary Information [file 41467_2021_21227_MOESM1_ESM.pdf]

SUPPLEMENTARY INFORMATION for

Positioning of nucleosomes containing γ-H2AX precedes active DNA demethylation and transcription initiation

Stephanie Dobersch[1, 2, 3], Karla Rubio[1, 2, 4, 5], Indrabahadur Singh[2, 6], Stefan Günther[7, 8], Johannes Graumann[9], Julio Cordero[10, 11], Rafael Castillo-Negrete[1, 2], Minh Bao Huynh[12], Aditi Mehta[2, 13], Peter Braubach[14, 15], Hector Cabrera-Fuentes[16-19], Jürgen Bernhagen[20, 21], Cho-Ming Chao[22, 23], Saverio Bellusci[22-25], Andreas Günther[24-27], Klaus T Preissner[16, 24], Sita Kugel[3], Gergana Dobreva[10, 11], Malgorzata Wygrecka[16, 24, 25, 28], Thomas Braun[8, 24], Dulce Papy-Garcia[12] and Guillermo Barreto[1, 2, 4, 5, 24, 25, *]

This PDF file includes: Supplementary Introduction, Supplementary Results. Supplementary Discussion, Supplementary Figures 1 to 10, Supplementary Table 1, Supplementary Table 2 and Supplementary References

Other Supplementary Materials for this manuscript include the following:

Source Data file 01: The source data for all the plots including the values for statistical significance and the implemented statistical tests in all plots presented in the article.

Source Data file 02: The uncropped pictures of all the blots presented in the main and Supplementary Figures.

Source Data file 03: Relevant instrumentation parameters used during mass spectrometry analysis were extracted using MARMoSET.

Source Data file 04: Data sheets containing ChIP-seq, RNA-seq and mass spectrometry based proteomic presented in Figures 1c, 3a, 3b, 4b, 8a, 8b, 9a and 9b. See Source Data file01.

Supplementary Introduction

Histone variants are strongly conserved among species, suggesting that they arose at an early stage in evolution.

HMG proteins are divided into three families based on their DNA binding domains: HMGA (containing AT-hooks), HMGB (containing HMG-boxes) and HMGN (containing nucleosomal binding domains)[1, 2].

Under normal circumstances fibroblasts are important for wound healing and connective tissue production. However, in the fibrotic lung their function is impaired resulting in formation of fibroblastic foci, which consist of highly proliferative fibroblasts, immune cells, and excessive extracellular matrix (ECM) protein deposition, such as fibronectin (FN1) and collagen (COL1A1)[3]. Consequently, these processes result in disproportionate levels of scar tissue, alterations of the lung epithelium structure and loss of the gas exchange function of the lung.

Supplementary Results

HMGA2-lyase activity is required for pH2A.X deposition and solving of R-loops

It has been reported that HMGA2 efficiently cleaves DNA generating single-strand breaks[4]. The arginine residues of the C-terminal AT-hook motif are crucial for this intrinsic lyase activity of HMGA2 (Supplementary Fig. 6a, top). Thus, to further elucidate the molecular mechanism of

HMGA2-mediated transcription activation, a lyase-deficient HMGA2 mutant was generated by substituting these arginine residues by alanine (RΔA HMGA2; Supplementary Fig. 6a, bottom). Further, *Hmga2*-/- MEF were stably transfected with a tetracycline-inducible expression construct containing the cDNA of RΔA HMGA2-MYC-HIS. Doxycycline treatment of these stably transfected MEF induced the expression of RΔA HMGA2-MYC-HIS to similar levels as in MEF containing WT HMGA2-MYC-HIS (Supplementary Fig. 6b, top). Furthermore, analysis of the chromatin-bound sub-nuclear fraction of these cells (Supplementary Fig. 6b, bottom) demonstrated that the R to A mutations did not significantly affect the binding of HMGA2 to chromatin. In addition, the loss of lyase activity in RΔA HMGA2-MYC-HIS was confirmed by monitoring DNA damage using Comet Assays[5] (Supplementary Fig. 6c) or by monitoring HMGA2-DNA complexes on dot blots (Supplementary Fig. 6d) after trapping experiments[4] using $NaCNBH_3$ as reducing agent to trap the Schiff base intermediates of the lyase reaction mediated by HMGA2. Furthermore, to demonstrate that the R to A mutations in the third AT-hook domain of HMGA2 do not affect the interaction with the FACT complex, we performed Co-IP of nuclear protein extracts from the stably transfected *Hmga2*-/- MEF described above (Supplementary Fig. 6e). IP using nuclear extracts and MYC-specific antibodies specifically co-precipitated both components of the FACT complex, SUPT16 and SSRP1, without significant differences between WT and RΔA HMGA2-MYC-HIS. Moreover, we analyzed the promoters of *Gata6, Mtor* and *Igf1* (Supplementary Fig. 6f) by ChIP using chromatin isolated from the same cells used for the Co-IP (Supplementary Fig. 6e). We found that the enrichment of WT and RΔA HMGA2-MYC-HIS was not significantly different at the *Igf1* promoter, whereas we observed an increased enrichment of RΔA HMGA2-MYC-HIS at the *Gata6* and *Mtor* promoters when compared to WT HMGA2-

MYC-HIS. These results indicate that the interaction with the FACT complex and the enrichment at the analyzed promoters was similar for WT and RΔA HMGA2.

Supplementary Discussion

Targeting the HMGA2-FACT-ATM-pH2A.X axis to specific loci

The presented experimental data robustly support the molecular mechanism proposed here. Nevertheless, this work should be considered as starting point of future projects that will elucidate exciting questions that remained open. For example, it is currently unclear how HMGA2 is targeted to specific promoters. One answer to this question might be the inducibility of specific genes in determined signaling pathways, including TGFB, IGF and WNT signaling pathways, especially since they have been related to HMGA2[6, 7, 8, 9]. However, this is just a partial answer, since there are genes that are inducible by these signaling pathways in an HMGA2-independent manner, as the ones shown in clusters 2 and 4 of Fig. 8a, as well as there are HMGA2 target genes that are not induced by these signaling pathways. Interestingly, we have observed that ncRNAs are transcribed from virtually all top 15% candidates (Supplementary Fig. 7a-b). In addition, 38.5% of the genes inside the top 15% candidates contain high GC skews that favor the formation of R-loops (Supplementary Fig. 7f-g). The DNA-ncRNA hybrids of the R-loops might be an excellent option to tether HMGA2 to specific promoters. Similarly, triple-helical RNA-DNA-DNA structures (triplex) at enhancers and promoters allow lncRNAs to recruit protein complexes to specific genomic regions and regulate gene expression[10, 11]. Interestingly, Summer and colleagues demonstrated in a seminal work that HMGA2 binds and efficiently cleaves ssDNA

containing abasic sites *in vitro*[4]. It will be the scope of our future work to determine whether the binding affinity and the cleavage efficiency of HMGA2 increase when the DNA substrate for the intrinsic lyase activity of HMGA2 is part of a DNA-RNA hybrid in an R-loop. Supporting this line of ideas, Arab and colleagues recently reported in a pioneering work that GADD45A preferentially binds DNA-RNA hybrids and R-loops rather than ssDNA, dsDNA or RNA[12]. Strikingly, we have shown that loss of HMGA2 lyase activity increased R-loop levels (Fig. 6c-d), while binding of GADD45A to R-loops was reduced (Fig. 7g-h), thereby supporting the hypothesis that single-strand breaks of the DNA moiety in the DNA-RNA hybrids are required for GADD45A binding.

Another open question, from the mechanistic point of view, is related to the origin of the abasic sites that are bound and cleaved by HMGA2. Since 38% of the genes inside the top 15% candidates contain GC skews and active DNA demethylation is part of the mechanism of transcription initiation proposed here, a plausible explanation for the generation of abasic sites is the participation of DNA glycosylases that removes the bases from 5-methycytosine (5mC) or thymine as deamination product of 5mC. In addition, HMGA2 has been shown to interact with APEX1 (apurinic/apyrimidinic site endonuclease 1)[4] and XRCC6 (X-ray repair cross-complementing protein 6, a ssDNA-dependent helicase also known as Ku70)[13], thereby supporting the involvement of the base excision repair (BER) machinery during HMGA2 function. It will be interesting to perform functional experiments demonstrating the requirement of the BER machinery during the mechanism of transcription initiation proposed here. Supporting these ideas, various reports demonstrate that transcription, DNA damage, and repair are mechanistically and functionally intertwined[14].

Opposite function of the HMGA2-FACT-ATM-pH2A.X axis and the MiCEE complex

We have recently reported a mechanism of transcription repression mediated by the multicomponent RNA–protein complex MiCEE[15]. In addition, we have shown that in IPF reduced levels of the micro RNA lethal 7d (*MIRLET7D*, also known as *let-7*) and hyperactive EP300 compromise the epigenetic gene silencing mediated by the MiCEE complex[16]. The results presented here strongly imply an opposite function of the MiCEE complex and the HMGA2-FACT-ATM-pH2A.X axis in the context of TGFB1 signaling and IPF. Supporting this line of evidence, it has been reported that *MIRLET7D* targets *HMGA2* mRNA, thereby preventing TGFB1-induced EMT and renal fibrosis[17]. Further, reduction of mature *MIRLET7* levels by the oncofetal protein LIN28B allows HMGA2 to drive an epigenetic program during pancreatic ductal adenocarcinoma (PDAC), one of the most lethal malignancies[18]. It will the scope of our future work to confirm the opposite function of the MiCEE complex and the HMGA2-FACT-ATM.pH2A.X axis within the context of fibrosis in different organs, including lung, kidney and liver.

Supplementary Figures 1 to 10

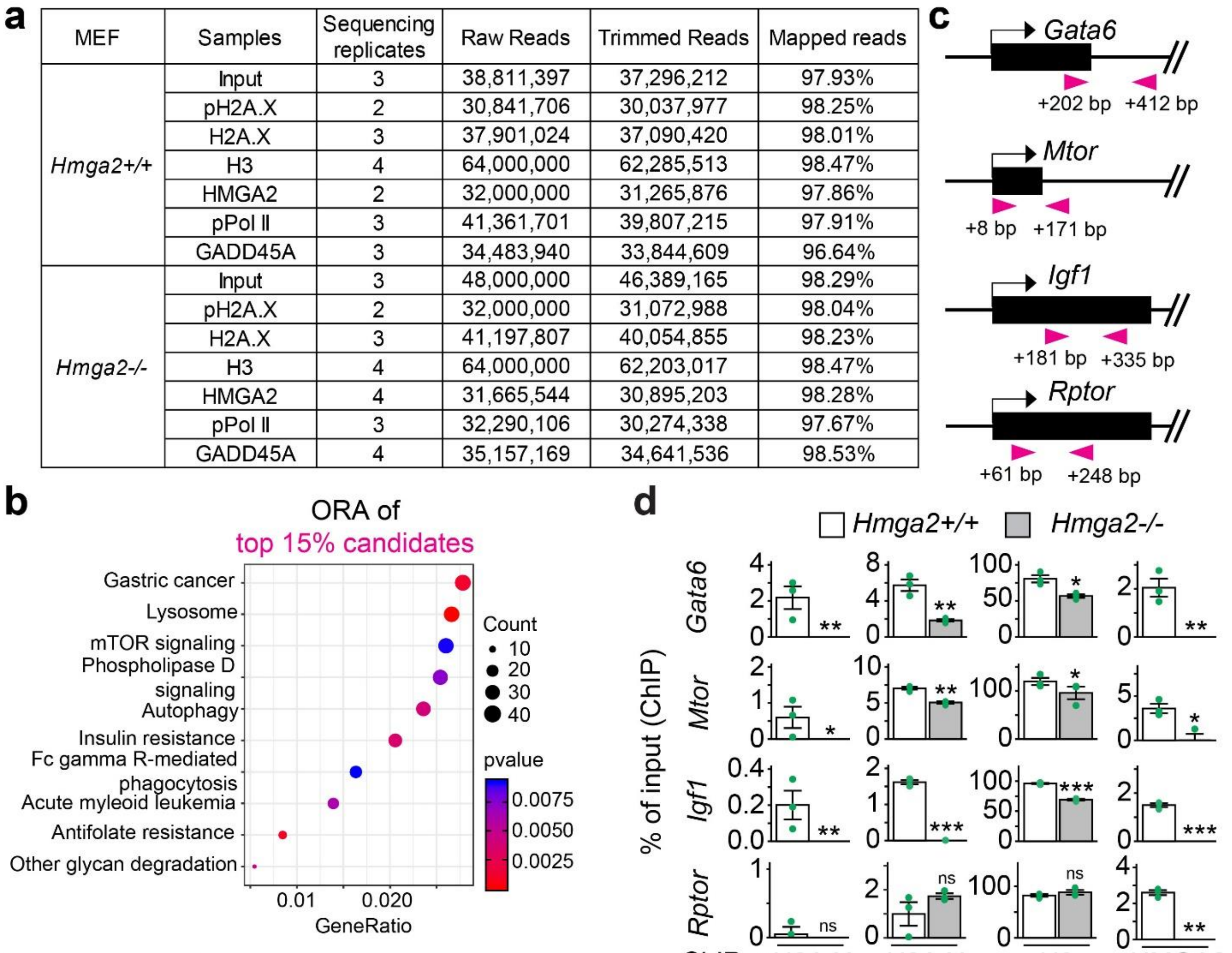

MEF	Samples	Sequencing replicates	Raw Reads	Trimmed Reads	Mapped reads
Hmga2+/+	Input	3	38,811,397	37,296,212	97.93%
	pH2A.X	2	30,841,706	30,037,977	98.25%
	H2A.X	3	37,901,024	37,090,420	98.01%
	H3	4	64,000,000	62,285,513	98.47%
	HMGA2	2	32,000,000	31,265,876	97.86%
	pPol II	3	41,361,701	39,807,215	97.91%
	GADD45A	3	34,483,940	33,844,609	96.64%
Hmga2-/-	Input	3	48,000,000	46,389,165	98.29%
	pH2A.X	2	32,000,000	31,072,988	98.04%
	H2A.X	3	41,197,807	40,054,855	98.23%
	H3	4	64,000,000	62,203,017	98.47%
	HMGA2	4	31,665,544	30,895,203	98.28%
	pPol II	3	32,290,106	30,274,338	97.67%
	GADD45A	4	35,157,169	34,641,536	98.53%

Supplementary Figure 1

Supplementary Figure 1: HMGA2 is required for pH2A.X deposition at TSS. (a) Description of the ChIP-seq data set supports the quality of the experiment. (b) KEGG-based enrichment analysis of top 15 % candidate genes using clusterProfiler. Fisher's Exact Test was used to calculate the *P* values. (c) Schematic representation of the 5’ genomic region of *Gata6*, *Mtor*, *Igf1* and *Rptor* showing exons (black boxes), introns (lines), arrows, direction of the genes and location of primer pairs (arrowheads) used for ChIP analysis. Number represents distance of the 5’ primer to the TSS. (d) ChIP analysis of *Gata6*, *Mtor*, *Igf1 and Rptor* TSS using specific antibodies as indicated and chromatin isolated from *Hmga2+/+* and *Hmga2-/-* MEF. In all plots, data are displayed as means

± s.e.m (n=3 biologically independent experiments); asterisks, P values after two-tailed t-Test, ***$P \leq 0.001$; **$P \leq 0.01$; *$P \leq 0.05$; ns, non-significant. Source data are provided as Source Data files 01 and 04.

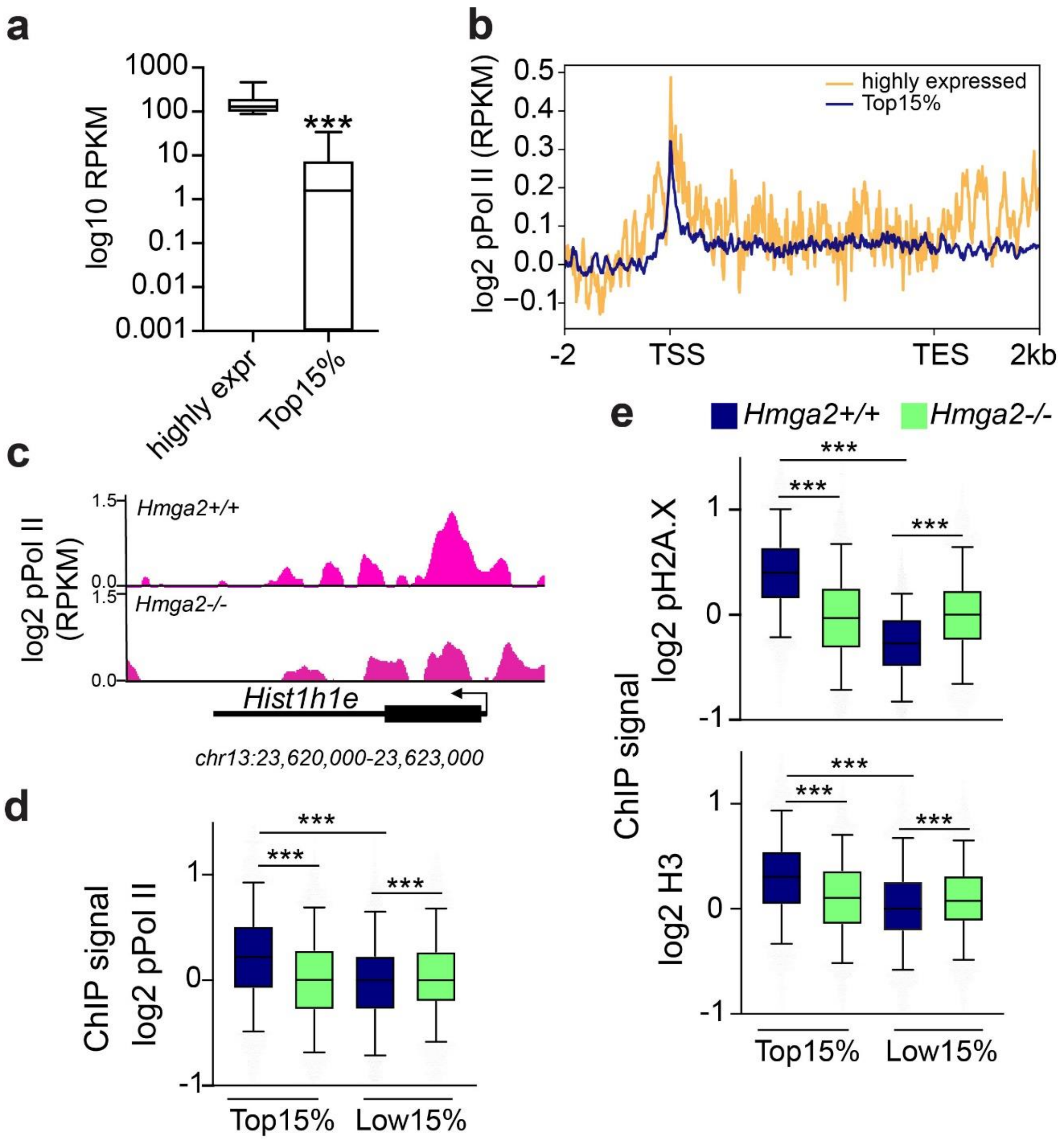

Supplementary Figure 2: Top 15% candidate genes show low basal transcriptional activity. (a) Box plot representing the basal transcription activity (as log10 RPKM) of the top 500 highly expressed genes (*n*=500 genes) versus the top 15% candidate genes (*n*=9522 genes) in *Hmga2+/+* MEF from one sequencing experiment. All box plots of this figure indicate median (middle line), 25th, 75th percentile (box) and 5th and 95th percentile (whiskers); asterisks, *P* values after two-

tailed Wilcoxon-Mann-Whitney test, *** $P \leq 0.001$. (b) Aggregate plots for phosphorylated serine 5 RNA polymerase II (pPol II) enrichment within the gene body ±2kb of the Top 500 highly expressed genes and the top 15% candidate genes in *Hmga2+/+* MEF. (c) Visualization of *Hist1h1e* gene locus using UCSC Genome Browser showing pPol II enrichment in *Hmga2+/+* and -/- MEF. *Hist1h1e* is found in the top 500 highly expressed genes. ChIP-seq reads were normalized using RPKM measure and are represented as log2 enrichment over their corresponding inputs. Images represent the indicated gene loci with their genomic coordinates. Arrows, direction of the genes; black boxes, exons; dotted squares, regions selected for single gene analysis. (d) Box plots of ChIP-seq-based pPol II enrichment analysis within the TSS + 0.5kb of the top 15% candidates or lowest 15% (Low) in *Hmga2+/+* and -/- MEF (*n*=9522 genes from one sequencing experiment). ChIP-seq reads were normalized using reads per kilobase per million (RPKM) measure and are represented as log2 enrichment over their corresponding inputs. (e). Box plots of ChIP-seq-based pH2A.X (top) and H3 (bottom) enrichment analysis within the TSS + 0.5kb of the top 15% candidates or lowest 15% (Low) in *Hmga2+/+* and -/- MEF (*n*=9,522 genes from one sequencing experiment). ChIP-seq reads were normalized using reads per kilobase per million (RPKM) measure and are represented as log2 enrichment over their corresponding inputs. Source data are provided as a Source Data file 01.

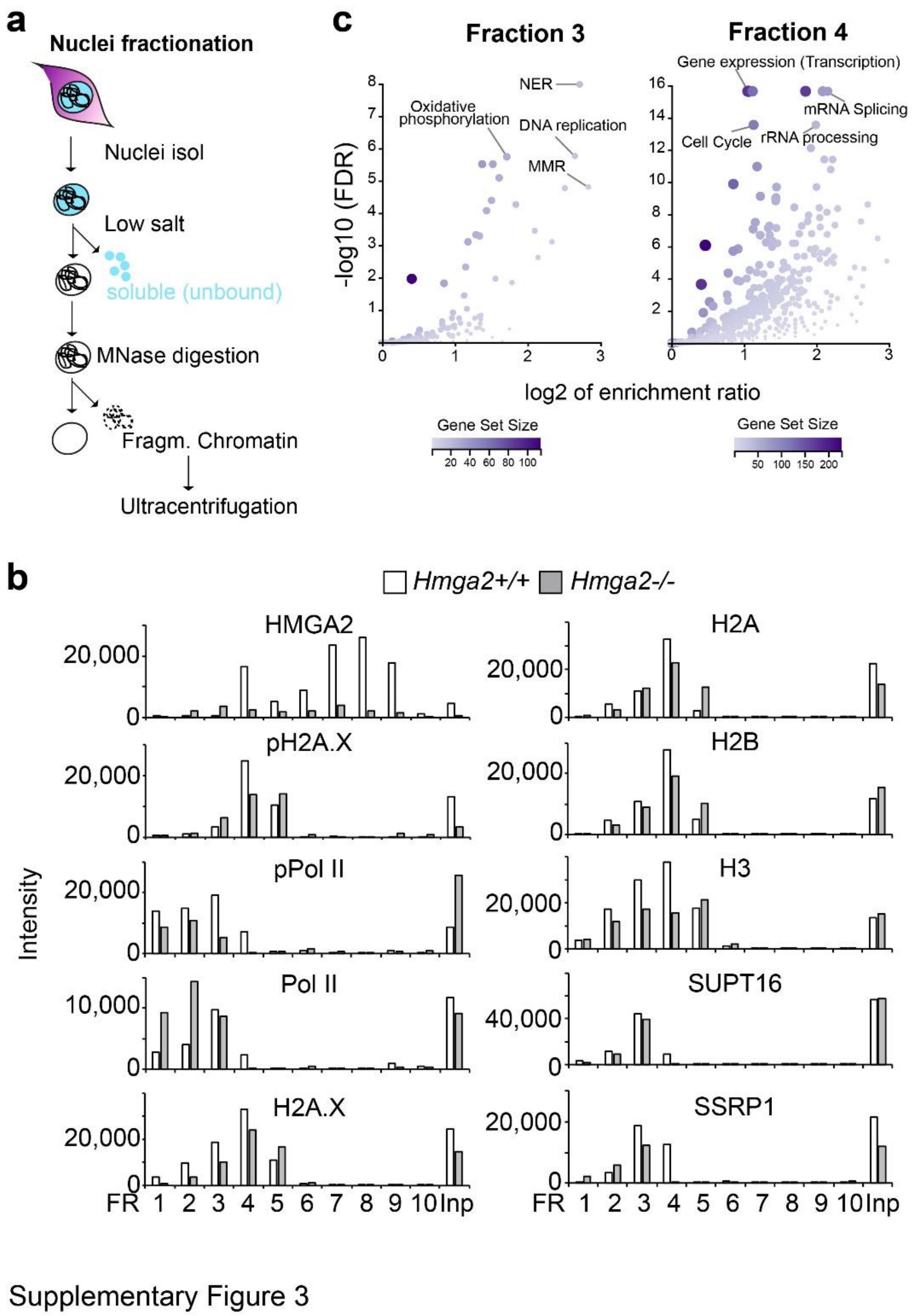

Supplementary Figure 3: Fractionation of native chromatin from *Hmga2*+/+ and -/- MEF. (a) Schematic representation of the experimental outline. Chromatin was prepared and fractionated by micrococcus nuclease digestion. Samples were loaded onto a sucrose gradient and were separated by ultracentrifugation. (b) Densitometry analysis of western blots shown in Fig. 4a and

4d. (c) Left, KEGG-based and, right, Reactome-based enrichment analysis of proteins showing significant enrichment in *Hmga2+/+* as compared to *Hmga2-/-* MEF in fraction 3 and 4 using WEB-based gene set analysis toolkit, respectively.

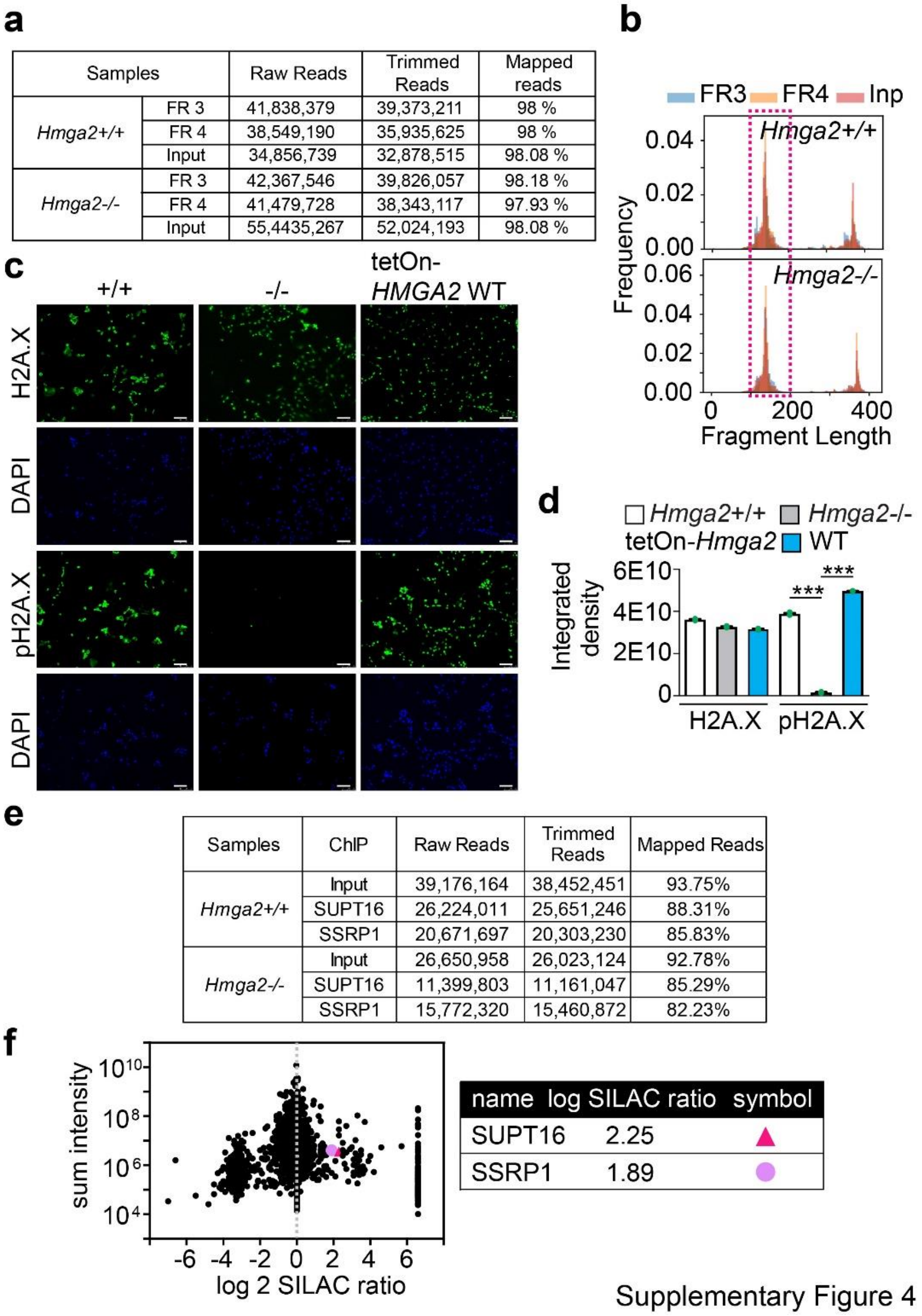

Samples		Raw Reads	Trimmed Reads	Mapped reads
Hmga2+/+	FR 3	41,838,379	39,373,211	98 %
	FR 4	38,549,190	35,935,625	98 %
	Input	34,856,739	32,878,515	98.08 %
Hmga2-/-	FR 3	42,367,546	39,826,057	98.18 %
	FR 4	41,479,728	38,343,117	97.93 %
	Input	55,4435,267	52,024,193	98.08 %

Samples	ChIP	Raw Reads	Trimmed Reads	Mapped Reads
Hmga2+/+	Input	39,176,164	38,452,451	93.75%
	SUPT16	26,224,011	25,651,246	88.31%
	SSRP1	20,671,697	20,303,230	85.83%
Hmga2-/-	Input	26,650,958	26,023,124	92.78%
	SUPT16	11,399,803	11,161,047	85.29%
	SSRP1	15,772,320	15,460,872	82.23%

name	log SILAC ratio	symbol
SUPT16	2.25	▲
SSRP1	1.89	●

Supplementary Figure 4: Ectopic expression of HMGA2 in *Hmga2-/-* MEF restores pH2A.X levels. (a) and (e) Description of the MNase-seq (a), SUPT16 and SSRP1 ChIP-seq (e) data set supports the quality of the experiments. (b) Frequency of fragment length distribution of reads obtained from fraction 3 and 4 after paired-end sequencing. Reads with a length of 100 to 200 bp

were selected for further analysis. (c) Representative pictures of confocal microscopy after immunostaining using H2A.X or pH2A.X-specific antibodies in *Hmga2+/+*, *Hmga2-/-* MEF and *Hmga2-/-* MEF that were stably transfected with a tetracycline-inducible expression construct (tetOn) for WT *Hmga2-myc-his*. MEF were treated with doxycycline for 4 h. DAPI, nucleus. Scale bars, 75 µm. (d) Quantification of the integrated density of immunofluorescence images form (c). Data are displayed as means ± s.e.m (n=3 independent experiments); asterisks, P values after two-tailed t-Test, ***$P \leq 0.001$. **(**f) HMGA2-interacting proteins identified by mass spectrometry analysis published by[7]. Scatter plot between SILAC ratios and peak intensities (top); selected proteins with corresponding log2 SILAC ratios of SUPT16 and SSRP1 (bottom).

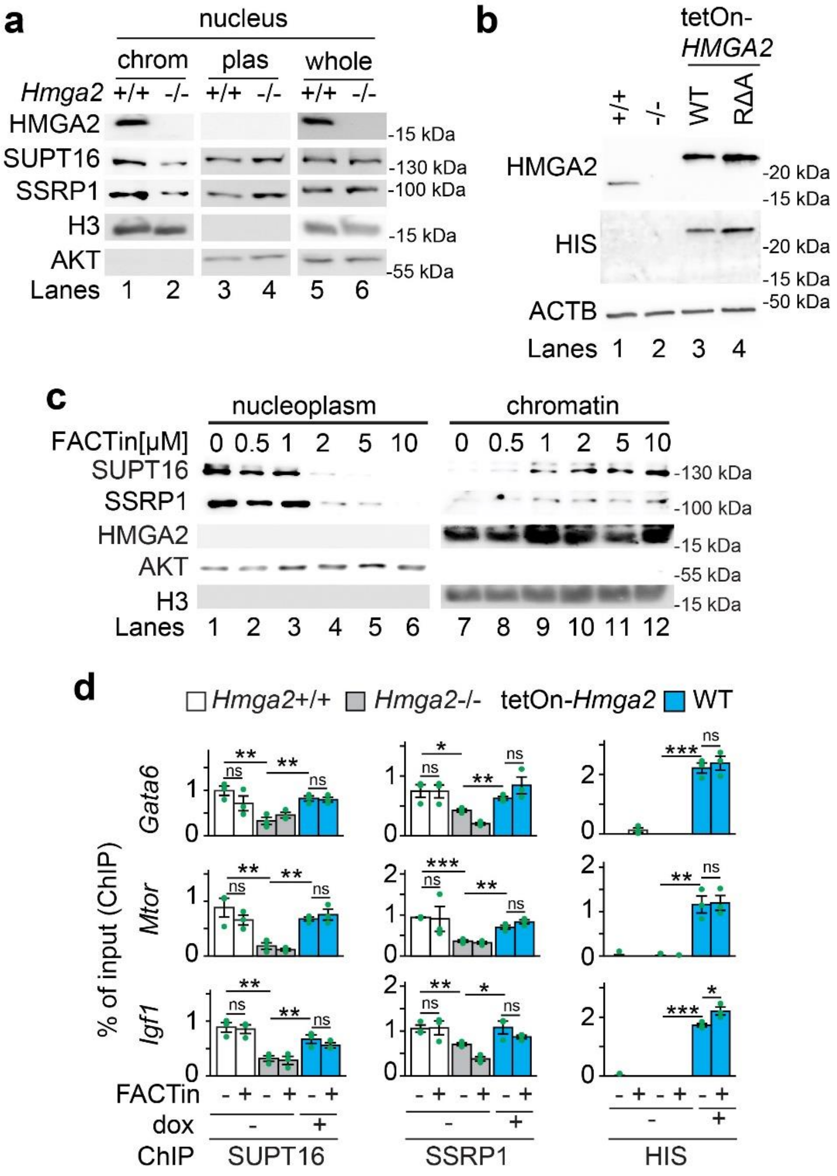

Supplementary Figure 5: HMGA2 and FACTin increases FACT complex binding to chromatin. (a) WB analysis of chromatin (chr), nucleoplasm (nuc pl) and nuclear lysates (nuc lys) from *Hmga2+/+* and *Hmga2-/-* MEF using the indicated antibodies. H3 and AKT were used as markers for chromatin and nucleoplasm, respectively. (b) WB analysis of nuclear lysates from *Hmga2+/+*, *Hmga2-/-* MEF, as well as *Hmga2-/-* MEF that were stably transfected with a tetracycline-inducible expression construct (tetOn) either for WT *Hmga2-myc-his* or the lyase-deficient mutant RΔA *Hmga2-myc-his*. MEF were treated with doxycycline for 24 h and lysates

were probed for the indicated antibodies. (c) *Hmga2+/+* MEF were treated for 2 h with the indicated concentrations of CBLC000 trifluoroacetate (FACTin). Afterwards, nucleoplasm and chromatin were isolated and analyzed by WB using the noted antibodies. AKT and H3 were used as nucleoplasm and chromatin markers, respectively. WB images presented in panels (a) to (c) are representative for three independent experiments, respectively. (d) ChIP-based promoter analysis of *Gata6*, *Mtor* and *Igf1* using the indicated antibodies and chromatin from *Hmag2+/+*, *Hmag2-/-* MEF, as well as *Hmga2-/-* MEF that were stably transfected with a tetracycline-inducible expression construct (tetOn) for WT *Hmga2-myc-his*. MEF were treated with doxycycline and FACT inhibitor (FACTin; CBLC000 trifluoroacetate) as indicated. Data are displayed as means ± s.e.m (n=3 independent experiments); asterisks, P values after one tailed t-Test, ***$P \leq 0.001$; **$P \leq 0.01$; *$P \leq 0.05$; ns, non-significant. Source data are provided as a Source Data file 01 and 02.

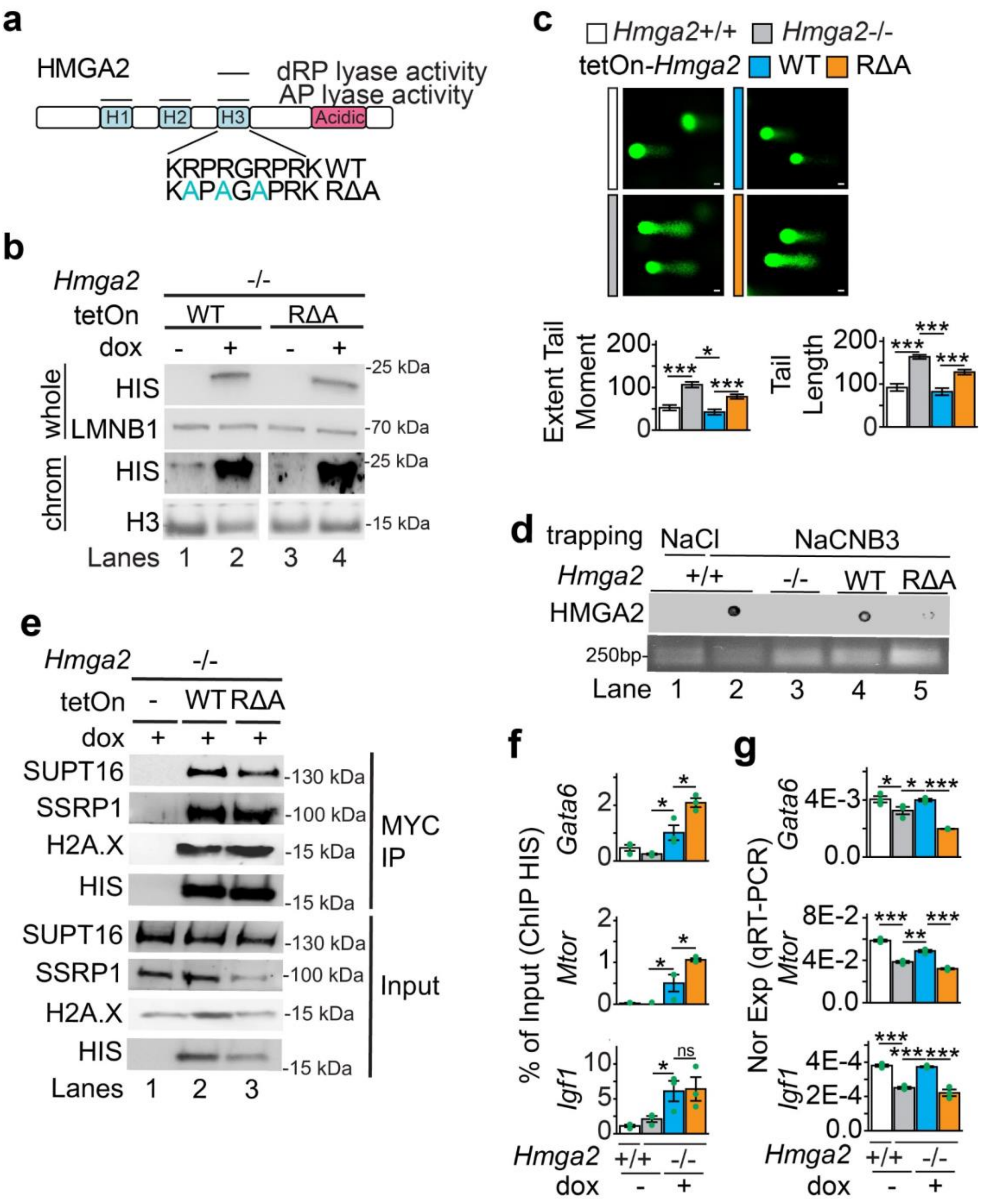

Supplementary Figure 6: Lyase activity-dependent pH2A.X deposition is required for pPol II enrichment and gene transcription. (a) Schematic representation of HMGA2 comprising AP lyase activity within the three AT-hook domains (H1-3) and a 5'-desoxyribose phosphate (dRP) lyase activity. Arginine (R) residues were mutated to alanines to abolish lyase activity in the third

hook domain (RΔA mutant). (b) WB analysis of nuclear lysate (top) and chromatin (bottom) of *Hmga2-/-* MEF that were stably transfected with a tetracycline-inducible expression construct (tetOn) either for WT *Hmga2-myc-his* or the lyase-deficient mutant RΔA *Hmga2-myc-his* using the indicated antibodies. LMNB1 and H3 were used as a loading control. (c) Top, representative images of comet assay using *Hmga2+/+*, *Hmga2-/-* MEF, as well as *Hmga2-/-* MEF that were stably transfected with a tetracycline-inducible expression construct (tetOn) either for WT *Hmga2-myc-his* or the lyase-deficient mutant RΔA *Hmga2-myc-his*. Cells were treated for 6 h with doxycycline. Bottom, quantification of extent tail moment and tail length in imaged MEF (*n*=28 for *Hmga2+/+*, *n*=24 for *Hmga2-/-*, *n*=27 for *Hmga2-/- tetOn WT Hmga2-myc-his* and *n*=32 for *Hmga2-/-* RΔA *Hmga2-myc-his* cells examined from one representative experiment). Scale bars, 50 µm. (d) Dot-blot confirming the impaired lyase activity of the RΔA mutant. A control for the amount of DNA loaded is shown below the blot. (e) Immunoprecipitation of HMGA2 from nuclear protein extracts of *Hmga2-/-* MEF, as well as *Hmga2-/-* MEF that were stably transfected with a tetracycline-inducible expression construct (tetOn) either for WT *Hmga2-myc-his* or the lyase-deficient mutant RΔA *Hmga2-myc-his* using MYC-coated magnetic beads. Co-immunoprecipitated proteins were analyzed by WB using the indicated antibodies. Input (Inp), 4% of material used for the IP. (f) ChIP analysis of HMGA2 targets using a HIS-tag specific antibody with chromatin isolated from *Hmga2+/+*, *Hmga2-/-* MEF, as well as *Hmga2-/-* MEF that were stably transfected with a tetracycline-inducible expression construct (tetOn) either for WT *Hmga2-myc-his* or the lyase-deficient mutant RΔA *Hmga2-myc-his*. Cells were treated for 4h with doxycycline as indicated. (g) QRT-PCR analysis using *Hmga2+/+*, *Hmga2-/-* MEF, as well as *Hmga2-/-* MEF that were stably transfected with a tetracycline-inducible expression construct (tetOn) either for WT *Hmga2-myc-his* or the lyase-deficient mutant RΔA *Hmga2-myc-his*. Cells

were treated for 6h with doxycycline. Images in panels (b) to (e) are representative for three independent experiments. In all plots, data are displayed as means ± s.e.m (n=3 independent experiments); asterisks, P values after two-tailed (in c) or one-tailed (in f and g) t-Test, ***$P \leq 0.001$; **$P < 0.01$; *$P \leq 0.05$; ns, non-significant. Source data are provided as a Source Data file 01 and 02.

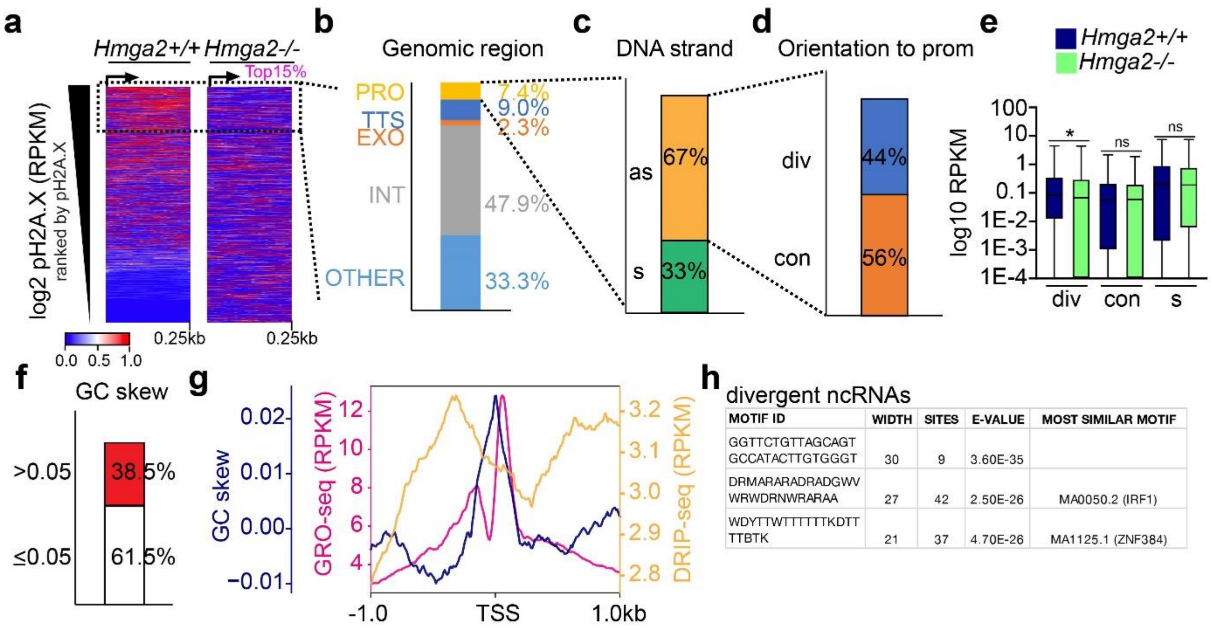

Supplementary Figure 7: Characterization of ncRNAs transcribed from the top 15% HMGA2 candidate genes. (a) Heat map for pH2A.X enrichment at the TSS +0.25kb of UCSC Known Genes in *Hmga2+/+* and *Hmga2-/-* MEF. Genes were ranked by pH2A.X enrichment in *Hmga2+/+* MEF. Doted square, the top 15% ranked genes. (b) Distribution of ncRNA loci in close proximity to the top 15 % candidate gene bodies ± 5kb; PRO, promotor; TTS, transcription termination site; EXO, exon; INT, intron. (c) Analysis of the orientation of ncRNAs towards the corresponding top 15% candidate genes; as, antisense; s, sense. (d) Subclassification of antisense ncRNAs; div, divergent; con, convergent. (e) Box plot representing the basal transcription activity (as log10 RPKM) of ncRNA in *Hmga2+/+* and *Hmga2-/-* MEF. Box plots indicate median (middle line), 25th, 75th percentile (box) and 5th and 95th percentile (whiskers); *n*=621 ncRNAs for divergent (div); *n*=780 ncRNAs for convergent (con); *n*=698 ncRNAs for sense (S) from one sequencing experiment; asterisks, *P* values after two-tailed Wilcoxon-Mann-Whitney test, * $P \leq 0.05$; ns, non-significant. Source data are provided as a Source Data file 01. (f) Bar chart highlighting the distribution of the top 15 % genes with a high GC skew (>0.05). Values represent

the average score in the TSS+250bp region. (g) Aggregate plot representing the global distribution of nascent RNA (GRO-seq) in WT MEF of the top 15 % candidate genes with associated antisense ncRNA, GC skew and RNA-DNA hybrids (DRIP-seq) in NIH/3T3 murine fibroblasts. Reads were normalized using an RPKM measure. TSS, transcription start site; kb, kilobases). (h) Table of the enriched transcription factor motifs in divergent ncRNA sequences using HOMER.

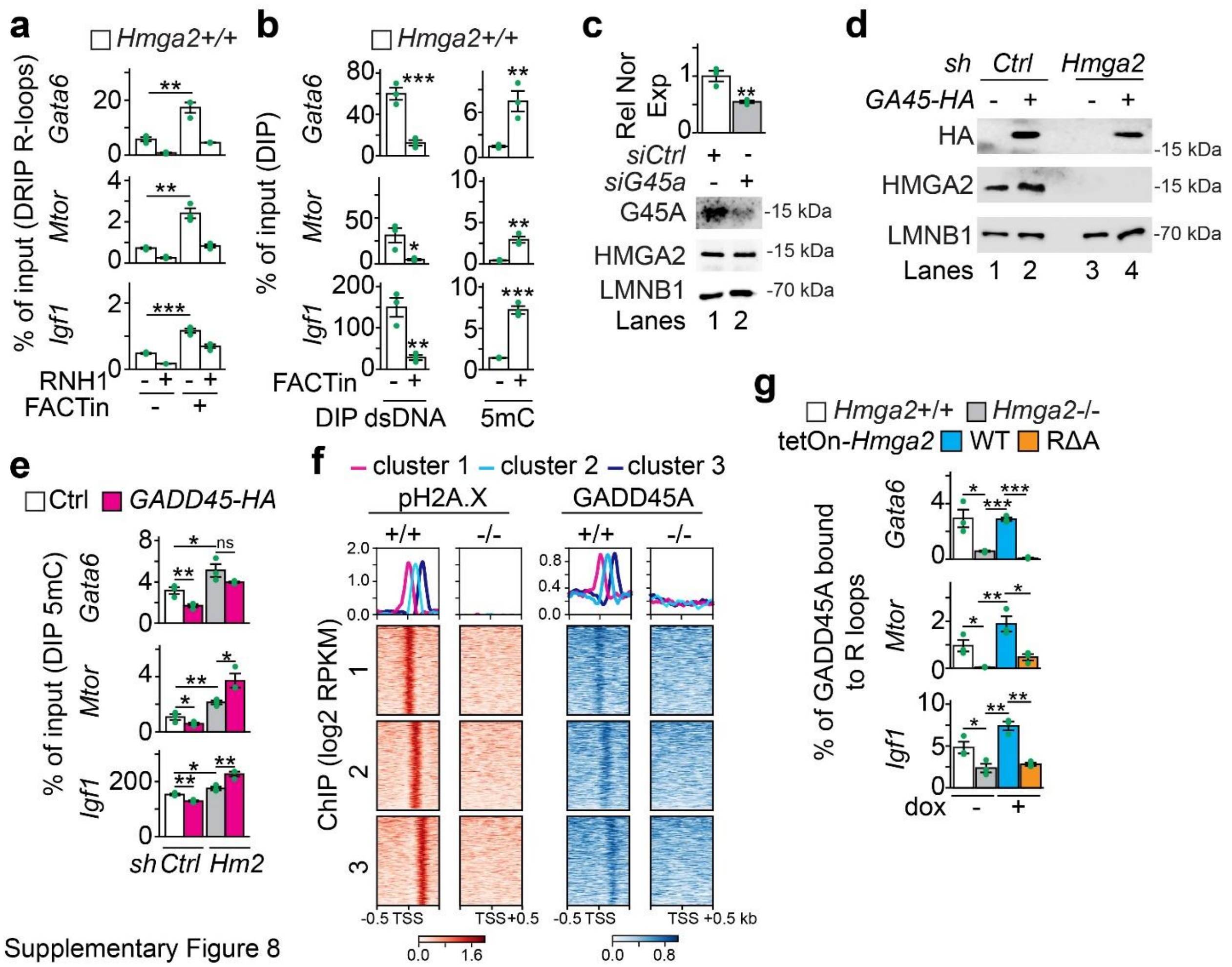

Supplementary Figure 8

Supplementary Figure 8: R-Loops correlate with pH2A.X enrichment and GADD45A-induced DNA demethylation. (a) Analysis of R-loops at the TSS of the selected HMGA2 target genes in *Hmga2+/+* MEF treated for 4 h with FACTin as indicated. (b) Analysis of dsDNA and 5mC as in a. (c) qRT-PCR analysis and WB for *Gadd45a* expression in *Hmga2+/+* MEF after siRNA-mediated KD. (d) and (e) WB analysis (d) and DIP for 5mC (e) after overexpression of *GADD45*A tagged with HA in MLE-12 cells that were stably transfected either with a control (scramble, *scr*) or an *Hmga2*-specific short hairpin DNA (*sh*) construct. WB images in (d) are representative for two independent experiments. (f) Aggregate plot and heat maps for pH2A.X and GADD45A enrichment at the TSS ± 0.5kb of the top 15 % candidate genes in *Hmga2+/+* and

Hmga2-/- MEF within the same clusters identified in Fig. 2e. (g) DRIP followed by sequential ChIP for GADD45A in *Hmga2+/+*, *Hmga2-/-* MEF, as well as *Hmga2-/-* MEF that were stably transfected with a tetracycline-inducible expression construct (tetOn) either for WT *Hmga2-myc-his* or the lyase-deficient mutant RΔA *Hmga2-myc-his*. Cells were treated for 4h with doxycycline. In all plots, data are displayed as means ± s.e.m (*n*=3 biologically independent experiments); asterisks, *P* values after two-tailed *t*-Test, ***$P \leq 0.001$; **$P \leq 0.01$; *$P \leq 0.05$; ns, non-significant. Source data are provided as a Source Data file 01 and 02.

a

MEF	Treatment	Raw reads	Equal reads	Trimmed reads	Mapped reads
Hmga2+/+	Ctrl	75,412,898	65,000,000	64,594,314	92.85%
	TGFB1	70,462,725	65,000,000	64,700,273	94.05%
Hmga2-/-	Ctrl	68,386,131	65,000,000	64,662,069	93.38%
	TGFB1	68,709,598	65,000,000	64,672,562	93.75%

b

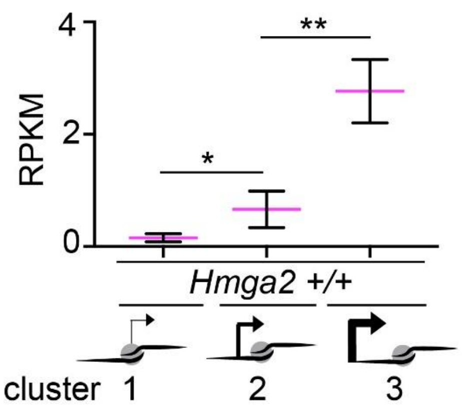

d

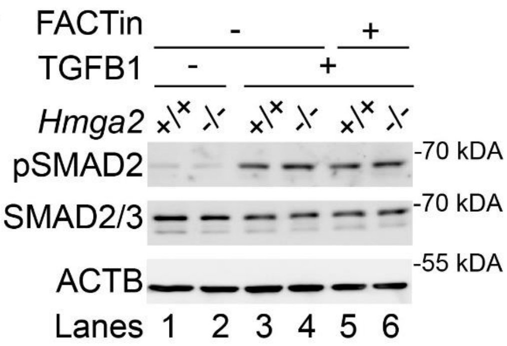

c

MEF	Treatrment	ChIP	Raw Reads	Trimmed Reads	Mapped reads
Hmga2+/+	Ctrl	Input	39,176,164	38,452,451	93.75%
	TGFB1	Input	42,007,733	41,252,957	94.12%
Hmga2-/-	Ctrl	Input	26,650,958	26,023,124	92.78%
	TGFB1	Input	39,046,794	38,150,975	93.79%
Hmga2+/+	Ctrl	pH2A.X	23,489,292	23,113,165	90.51%
	TGFB1	pH2A.X	31,544,791	30,968,223	89.42%
Hmga2-/-	Ctrl	pH2A.X	42,390,323	41,709,789	91.32%
	TGFB1	pH2A.X	28,777,086	28,274,372	91.69%

Supplementary Figure 9: GADD45A is required for TGFB1-enhanced active DNA methylation. (a) and (c) Descriptions of the RNA-seq data and TGFB1 ChIP-seq set support the quality of the experiments. (b) TGFB1-inducible genes in WT MEF were selected from Figure 3b and their expression was analyzed in *Hmga2+/+* and *Hmga2-/-* MEF measured by RPKM. Data are displayed as means ± s.e.m (*n*=36 genes in position cluster 1, n=21 genes in position cluster 2 and n=35 genes in position cluster 3 from one sequencing experiment); asterisks, *P* values after one-tailed Wilcoxon-Mann-Whitney test, ** $P \leq 0.01$; * $P \leq 0.05$. (d) WB analysis of phosphorylated Smad2 (Ser465/467), pSMAD) and total SMAD2/3 of *Hmga2+/+* and *Hmga2-/-*

MEF treated with TGFB1 alone or in combination with FACTin. Images are representative for three independent experiments. Source data are provided as a Source Data file 01 and 02.

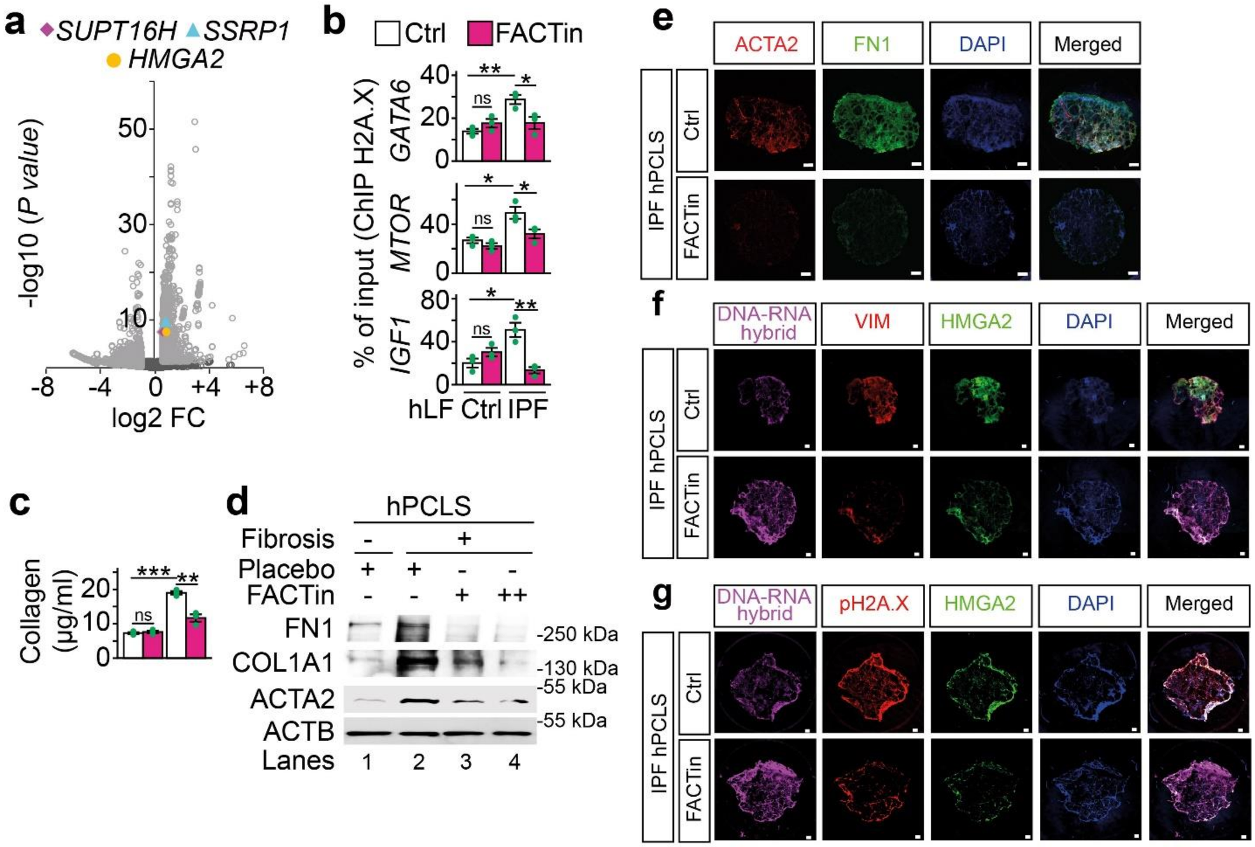

Supplementary Figure 10: FACTin reduces fibrotic markers and pH2A.X levels in PCLS. (a) Volcano plot representing the significance (−log10 *P* values after Wald t-test) vs. expression fold change (log2 expression ratio) between two Ctrl and two IPF patients. Fold changes of *SUPT16H*, *SSRP1* and *HMGA2* in IPF are highlighted. Light grey color marks genes with a FC>1.5 and *P*<0.05. (b) ChIP-based promoter analysis of selected HMGA2 target genes using H2A.X-specific antibody and chromatin from hLF treated as in Fig. 7d. (c) Quantification of collagen content in Ctrl and IPF hLF treated with Ctrl or FACTin for 24h. In all bar plots, data are shown as means ± s.e.m. (*n*=3 biologically independent experiments); asterisks, *P* values after two-tailed *t*-Test, *** $P \leq 0.001$; ** $P \leq 0.01$; * $P \leq 0.05$; ns, non-significant. (d) WB analysis of IPF markers in Ctrl and IPF human precision-cut lung slices (hPCLS) treated with FACTin using the indicated antibodies. (e) to (g) Representative pictures of confocal microscopy after immunostaining using

ACTA2, FN1, S9.6, VIM, HMGA2 or pH2A.X -specific antibody in hPCLS from IPF patients. The hPCLS were treated as in Figure 9i. ACTA2, smooth muscle actin alpha 2; FN1, fibronectin; VIM, vimentin; DAPI, nucleus. Scale bars, 500 μm. Images presented in panels (d) to (g) are representative for three independent experiments. Source data are provided as a Source Data file 01 and 02.

Supplementary Table 1: Primer sequences and sequences of shRNA constructs

Gene		Primer Sequence (5`-3`)	
Cloning			
Hmga2	Cloning	Forward	AAGCTAGCATGAGCGCACGCGGTGAGGGCG
		Reverse	AAGGATCCTCAATGGTGATGGTGATGATGACCGGTA
Hmga2 R75A	Mutagenesis	Forward	GGAGAAAAAGCGCCAAGAGGCAGACCTAGGAAATGG
		Reverse	CCATTTCCTAGGTCTGCCTCTTGGCGCTTTTTCTCC
Hmga2 R75A, R77A	Mutagenesis	Forward	GGAGAAAAAGCGCCAGCAGGCAGACCTAGGAAATGG
		Reverse	CCATTTCCTAGGTCTGCCTGCTGGCGCTTTTTCTCC
Hmga2 R75A, R77A, R79A	Mutagenesis	Forward	GGAGAAAAAGCGCCAGCAGGCGCACCTAGGAAATGG
		Reverse	CCATTTCCTAGGTGCGCCTGCTGGCGCTTTTTCTCC
Gene expression			
Hmga2	qPCR	Forward	GCAGCAGCAAGAGCCAACCTG
		Reverse	GTCTCTTCAGTCTCCTGAGCA
Gata6	qPCR	Forward	GCCCCGAAACGCTTCGGCAG
		Reverse	TTTGGGGTGGCCTCGGCTCT
Mtor	qPCR	Forward	CGTCACAATGCAGCCAACAA
		Reverse	TGCCTTTCACGTTCCTCTCC
Igf1	qPCR	Forward	GAGCTTACTGGCGGCTGCTT
		Reverse	GGCAAGTGACAGAGGCAGCA
Rptor	qPCR	Forward	GTGCCGAGCATATGCCAAAG
		Reverse	TCCCACTCGTGCAGTTTCTC
Tuba1a	qPCR	Forward	CCGCGAAGCAGCAACCAT
		Reverse	CCAGGTCTACGAACACTGCC
Gadd45a	qPCR	Forward	TGCTGCTACTGGAGAACGAC
		Reverse	TCCATGTAGCGACTTTCCCG
GATA6	qPCR	Forward	TTGACTGACGGCGGCTGGTG
		Reverse	CTCCCGCGCTGGAAAGGCTC
MTOR	qPCR	Forward	GTGTGTGGAGACAGGGGCTT
		Reverse	TCTGGGTCTTGGGCATGTCG
IGF1	qPCR	Forward	GTGTGTGGAGACAGGGGCTT

		Reverse	TCTGGGTCTTGGGCATGTCG
FN1	qPCR	Forward	CATGAAGGGGGTCAGTCCTA
		Reverse	CTTCTCAGCTATGGGCTTGC
COL1A	qPCR	Forward	AAGCGAGGAGCTCGAGGTGAAC
		Reverse	TTGGCACCAGGCAGACCAGCTT
HPRT1	qPCR	Forward	TTTGCTTTCCTTGGTCAGGCAGT
		Reverse	CGTGGGGTCCTTTTCACCAGCA
ACTA2	qPCR	Forward	TGAGCTTCGTGTTGCCCCTG
		Reverse	GTAACGAAGGAATAGCCACGC
Chromatin-Immunoprecipitation Assay			
Igf1 TSS	ChIP	Forward	GTGAATCGGCTGCTGCTTGC
		Reverse	GGCAGAACATAGACAGCGGCA
Gata6 TSS	ChIP	Forward	GTCGCGGCCGTTCTTCTCGC
		Reverse	GGTGGAGGCTGGTCCGGAGT
Mtor TSS	ChIP	Forward	CTAGAAGACAGCGGGGAAGG
		Reverse	CACACTGGCTGGGAGTCTG
Rptor TSS	ChIP	Forward	TTTGGCTTATTGGACGCGCC
		Reverse	GAGTCCCGATACACACGCGA
IGF1 TSS	ChIP	Forward	AGATAGAGCCTGCGCAATGGA
		Reverse	CGAGGAGGACATGGTGTGCA
GATA6 TSS	ChIP	Forward	TTCCCTCCTTCCCTCCGGGC
		Reverse	TAACTACCGGCTCCCGCCCC
MTOR TSS	ChIP	Forward	TGTCGATTGGTCCTCAGGGC
		Reverse	TGGACATTACGCCGCCCTAG
Nick assay			
Gata6 nick	QPCR	Fwd flank	ACATCGCTCGAGGTTCGGTT
		Rwd flank	ACACACAGACCCAGGCAAGATA
		Sense nick	GGTGTCCGGTCCTTCGCTTTA
		Antisense nick	TAAAGCGAAGGACCGGACAC
Mtor nick	QPCR	Fwd flank	TGGTTGTAGCGCTTACGA

		Rwd flank	GAGAAGGAGACCTAAATGCTC
		Sense nick	AGTCTGGGGCCTGGAGT
		Antisense nick	ACTCCAGGCCCCAGACT
Igf1 nick	QPCR	Fwd flank	TAAGGCTCTGCTCTTAGACTC
		Rwd flank	CAACTGCCTCCAAGGAGC
		Sense nick	TTGCCTCTGGCTCTGTCG
		Antisense nick	CGACAGAGCCAGAGGCAA
Rptor nick	QPCR	Fwd flank	AGTCCCGATAGTCTTAT
		Rwd flank	TCTACGGCGCGTCCAATAA
		Sense nick	TAACTGAACGGACATAGT

Vector name		Target sequence
pLKO.1-scrambled	Scrambled	5’-GCAAGCTGACCCTGAAGTTCAT-3’
pLKO.1-shHmga2	TRCN0000126045	5′-GCCACAACAAGTCGTTCAGAA-3′

Supplementary Table 2: Key Resources Table

REAGENT or RESOURCE	SOURCE	IDENTIFIER
**Antibodies**		
5mC	Abcam	ab10805
ACTA2	Sigma	A5228
ACTB	Sigma	A5316
AKT	Cell Signaling	9272S
AlexaFluor488 goat anti-rabbit	Invitrogen	A11008
AlexaFluor555 donkey anti-goat	Invitrogen	A21432
AlexaFluor594 goat anti-mouse	Invitrogen	A11005
COL1A1	Sigma	C2456
dsDNA	Abcam	ab27156
FN1	Millipore	AB2033
Gadd45a	Santacruz	sc-797<br>sc-6850
HA-tag	Santa Cruz	#sc-805
Histone H3 (ChIP Grade)	Abcam	ab1791
Histone H2A.X	Millipore	07-627
H2A.XS139ph	Millipore	05-636<br>07-164
Histone H2A	Abcam	#18255
Histone H4	Abcam	#7311
Histone H2B	Abcam	
His-tag	Abcam	ab9108
HMGA2	Abcam<br>Santa Cruz<br>CST	ab41878<br>sc- 30223<br>8179S
LMNB1	Santa Cruz	sc-6216
Mouse Control IgG	Santa Cruz Biotechnology, INC.	sc-2025
Donkey-anti mouse HRP	Jackson	715-035-150
SMAD2/3	Cell Signaling	3102S
pSMAD2 (S465/467)	Cell Signaling	3101S
Rabbit Control IgG	Santa Cruz Biotechnology, INC.	sc-2027
DNA-RNA hybrid [S9.6]	Kerafast	ENH001

RNA polymerase II CTD repeat YSPTSPS (phospho S5) antibody (ChIP Grade)	Abcam	ab5408
RNA polymerase II CTD repeat YSPTSPS (ChIP Grade)	Abcam	ab26721
SMAD3	Abcam	ab408554
SMAD3 (phospho S423+S425)	Abcam	Ab52903
SPT16	Cell Signaling	12191
SSRP1	BioLegend	609710
TET1	Active Motif	61443
VIM	Cell Signaling	5741S
**Chemicals and Reagents**		
Protein A Agarose/Salmon Sperm DNA	Millipore	16-157
Bovine Serum Albumin (BSA)	Carl Roth	8076.1
Pierce™ Coomassie Plus (Bradford) Assay Kit	Thermo Fisher Scientific	23236
CBLC000 trifluoroacetate	Sigma Aldrich	SML1974
Chloroform	Carl Roth	3313.1
DMEM, high glucose, pyruvate	Thermo Fisher	11995065
DMEM F-12 Ham	Corning	10-092-CM
DMSO	Sigma Aldrich	D2438
DPBS	Thermo Fischer Scientific	14190250
Doxycycline	Sigma Aldrich	D9891
Dulbecco's medium : Ham's F12	Gibco	11320033
Dynabeads® Protein A	Invitrogen	10002D
Formaldehyde	Sigma Aldrich	252549
Gemcitabine	Sigma Aldrich	G6423
KU-55933	Calbiochem	118500-2MG
Lipofectamine 2000	Invitrogen	11668027
Lipofectamine 3000	Invitrogen	L3000008
Luria broth	Roth	X964.1
Luria broth agar	Roth	6671.1

4–20% Mini-PROTEAN® TGX™ Precast Protein Gels	Bio-Rad	4561095
Nuclease-free water	Thermo Fisher Scientific	AM9937
Penicillin Streptomycin	Thermo Fisher Scientific	10378016
Polyallomer centrifuge tube	Beckman	344059
Polybrene	Sigma Aldrich	TR-1003
Protease Inhibitor Cocktail Set I	Calbiochem	539131
Puromycin	Sigma Aldrich	P8833
siCtrl	Ambion	AM4611
siGadd45a	Ambion	AM16708
Protein G Sepharose 4 Fast Flow	GE Healthcare	17061801
Sodium Chloride	Carl Roth	9265.1
Sodium-cyanoborohydride	Sigma Aldrich	296945
HighPrep™ PCR Clean-up System	Magbio	AC-60250
STAGE tips	In-house produced from Empore Octadecyl C18 Extraction Disks (Supelco)	66883-U
Sucrose	Sigma Aldrich	S0389-1KG
Transforming Growth Factor Beta	Sigma Aldrich	T7039
TRIzol® Reagent	Thermo Fisher Scientific	15596018
Triton-X100	Sigma Aldrich	T8787
Ultra-pure Phenol: Chloroform	Invitrogen	15593031
**Critical Commercial Assays and Kits**		
660 nm protein assay	Pierce	1861426
6-plex tandem mass tags	Thermo Fisher Scientific	90063
Pierce™ Anti-c-Myc Magnetic Beads	Thermo Scientific	88842
Comet Assay	Abcam	ab238544
LDH Cytotoxicity Detection Kit	Roche	11 644 793 001
High-Capacity cDNA Reverse Transcription Kit	Thermo Fisher Scientific	4368814
Ovation Ultralow System V2	Nugen	0344NB

Power SYBR Green Master Mix	Thermo Fisher Scientific	4368708
QIAquick PCR purification kit	Qiagen	28106
SMARTer Stranded Total RNA Sample Prep Kit - HI Mammalian	Clontech	634875
TruSeq ChIP Library Preparation Kit	Illumina	IP-202-1012
Quantitative fluorimetric peptide assay	Pierce	23290
Qubit dsDNA HS Assay Kit	Thermo Fisher Scientific	Q32854
QuikChange II Site-Directed Mutagenesis Kit	Agilent	200523
WesternBright ECL detection solution	Biozym	541004
**Enzymes**		
BsrGI	New England Biolabs	R3575S
EcoRI	New England Biolabs	R3101S
HindIII-HF	New England Biolabs	R3104S
Lys-C	Wako Chemicals GmbH	129-02541
Micrococcus nuclease	New England Biolabs	M0247S
RNase A	Sigma Aldrich	70856-3
SspI	New England Biolabs	R3132S
Trypsin	Serva	37286.03
Proteinase K	Sigma Aldrich	70663<br><br>P2308
Oligo R3	Thermo Fisher Scientific	1-1339-06
XbaI	New England Biolabs	R0145S
**Deposited Data**		
RNA-Seq	This paper	GEO: GSE141266 [https://www.ncbi.nlm.nih.gov/geo/query/acc.cgi?acc=GSE141266]
Proteomics	This paper	PRIDE: PXD016586 [https://www.ebi.ac.uk/pride/archive/projects/PXD016586]
H2A.X, H3, pPolII and GADD45A ChIP-Seq	This paper	GEO: GSE141264 [https://www.ncbi.nlm.nih.gov/geo/query/acc.cgi?acc=GSE141264]
pH2A.X, HMGA2 ChIP-Seq	PMID: 26045162	GEO: GSE63861 [https://www.ncbi.nlm.nih.gov/geo/query/acc.cgi?acc=GSE63861]

MNase-Seq after Ultracentrifugation	This paper	GEO: GSE141265 [https://www.ncbi.nlm.nih.gov/geo/query/acc.cgi?acc=GSE141265]
DRIP-Seq in NIH/3T3 cells	PMID: 27373332	GEO: GSE70189 [https://www.ncbi.nlm.nih.gov/geo/query/acc.cgi?acc=GSE70189]
SSRP1, SPT16 and pH2A.X, ChIP-Seq with and without TGFB1 treatment	This paper	GEO: GSE141271 [https://www.ncbi.nlm.nih.gov/geo/query/acc.cgi?acc=GSE141271]
Nuclear RNA-seq in Ctrl and IPF hLF	PMID: 31110176	GEO: GSE116086 [https://www.ncbi.nlm.nih.gov/geo/query/acc.cgi?acc=GSE116086]
GRO-Seq	PMID: 28424523	GEO: GSE76303 [https://www.ncbi.nlm.nih.gov/geo/query/acc.cgi?acc=GSE76303]
**Experimental Models: Cell Lines**		
Hmga2+/+ Mouse Embryonic Fibroblasts (MEF), E15.5	PMID: 26045162	n/a
Hmga2-/- MEF, E15.5	PMID: 26045162	n/a
Hmga2-/- MEF tetOn-*Hmga2* WT	This paper	n/a
Hmga2-/- MEF tetOn-*Hmga2* RΔA	This paper	n/a
HEK293T	ATCC	ATCC® CRL-3216
Healthy human lung fibroblasts	PMID: 31110176	n/a
IPF-derived human lung fibroblasts	PMID: 31110176	n/a
MLE-12 sh*Scrambled*	This paper	n/a
MLE-12 sh*Hmga2*	This paper	n/a
**Experimental Models: Organisms/Strains**		
Top10 *E.coli*		
Ctrl and IPF-derived PCLS		
**Recombinant DNA**		
pCMVR8.74		Addgene: #22036
pCW-*Hmga2* WT	This paper	n/a
pCW-*Hmga2* RΔA	This paper	n/a
pcDNA3-HA+WT GADD45	PMID: 10973963	Addgene: #24929
pMD2.G		Addgene: #12259
pLKO-scrambled	This paper	n/a
pLKO-*shHmga2*	This paper	n/a

**Sequence-Based Reagents n/a**		
Primers for Cloning	Sigma Aldrich	See Supplementary Table 1
Primers for RT-qPCR	Sigma Aldrich	See Supplementary Table 1
Primers for ChIP- and DRIP-qPCR	Sigma Aldrich	See Supplementary Table 1
Primers for Nick-qPCR	Sigma Aldrich	See Supplementary Table 1
**Software and Algorithms**		
analyzeRepeats.pl	HOMER	http://homer.salk.edu/homer/ngs/analyzeRNA.html
annotatePeaks.pl	HOMER	http://homer.ucsd.edu/homer/ngs/annotation.html
Bedtools		https://bedtools.readthedocs.io/en/latest/
Bowtie2	PMID: 22388286	http://bowtie-bio.sourceforge.net/bowtie2/index.shtml
Deeptools	PMID: 27079975	https://deeptools.readthedocs.io/en/develop/
DEseq2	doi: 10.1186/s13059-014-0550-8.).	http://bioconductor.org/packages/devel/bioc/vignettes/DESeq2/inst/doc/DESeq2.html
FastQC		https://www.bioinformatics.babraham.ac.uk/projects/fastqc/
fgsea		https://www.bioconductor.org/packages/release/bioc/vignettes/fgsea/inst/doc/fgsea-tutorial.html
Hisat2	PMID: 25751142	https://ccb.jhu.edu/software/hisat2/index.shtml
ImageJ		https://imagej.nih.gov/ij/
MakeTagDirectories	HOMER	http://homer.ucsd.edu/homer/ngs/tagDir.html
MARMoSET	PMID: 31097673	https://github.molgen.mpg.de/loosolab/MARMoSET_C
MASS		https://www.rdocumentation.org/packages/MASS/versions/7.3-53
MaxQuant suite of algorithms (v. 1.6.5.0)	PMID: 19029910	https://maxquant.org/
MACS	PMID: 21633945	https://taoliu.github.io/MACS/
Morpheus		https://software.broadinstitute.org/morpheus/
RNA hybrid-online server	PMID: 15383676	https://bibiserv.cebitec.uni-bielefeld.de/rnahybrid/
RStudio: Integrated Development for R.	RStudio Team (2015)	http://www.rstudio.com/

Samtools		http://www.htslib.org
T-coffee	PMID: 17526519	http://tcoffee.crg.cat/
trimmomatic v0.32	PMID: 24695404	http://www.usadellab.org/cms/?page=trimmomatic
UCSC Genome Browser	Genome Bioinformatics Group	https://genome.ucsc.edu/
UCSC Table Browser	Genome Bioinformatics Group	https://genome.ucsc.edu/cgi-bin/hgTables
Webgestalt	PMID: 31114916	http://www.webgestalt.org/

Supplementary References

1. Ozturk N, Singh I, Mehta A, Braun T, Barreto G. HMGA proteins as modulators of chromatin structure during transcriptional activation. *Front Cell Dev Biol* **2**, 5 (2014).

2. Bustin M. Revised nomenclature for high mobility group (HMG) chromosomal proteins. *Trends in biochemical sciences* **26**, 152-153 (2001).

3. Barkauskas CE, Noble PW. Cellular mechanisms of tissue fibrosis. 7. New insights into the cellular mechanisms of pulmonary fibrosis. *American journal of physiology Cell physiology* **306**, C987-996 (2014).

4. Summer H, *et al.* HMGA2 exhibits dRP/AP site cleavage activity and protects cancer cells from DNA-damage-induced cytotoxicity during chemotherapy. *Nucleic acids research* **37**, 4371-4384 (2009).

5. Jachimowicz RD, *et al.* UBQLN4 Represses Homologous Recombination and Is Overexpressed in Aggressive Tumors. *Cell* **176**, 505-519 e522 (2019).

6. Singh I, *et al.* Hmga2 is required for canonical WNT signaling during lung development. *BMC biology* **12**, 21 (2014).

7. Singh I, *et al.* High mobility group protein-mediated transcription requires DNA damage marker gamma-H2AX. *Cell research* **25**, 837-850 (2015).

8. Li Z, *et al.* An HMGA2-IGF2BP2 axis regulates myoblast proliferation and myogenesis. *Developmental cell* **23**, 1176-1188 (2012).

9. Brants JR, Ayoubi TA, Chada K, Marchal K, Van de Ven WJ, Petit MM. Differential regulation of the insulin-like growth factor II mRNA-binding protein genes by architectural transcription factor HMGA2. *FEBS letters* **569**, 277-283 (2004).

10. Blank-Giwojna A, Postepska-Igielska A, Grummt I. lncRNA KHPS1 Activates a Poised Enhancer by Triplex-Dependent Recruitment of Epigenomic Regulators. *Cell reports* **26**, 2904-2915 e2904 (2019).

11. Kuo CC, *et al.* Detection of RNA-DNA binding sites in long noncoding RNAs. *Nucleic acids research* **47**, e32 (2019).

12. Arab K, *et al.* GADD45A binds R-loops and recruits TET1 to CpG island promoters. *Nature genetics* **51**, 217-223 (2019).

13. Sgarra R, *et al.* Interaction proteomics of the HMGA chromatin architectural factors. *Proteomics* **8**, 4721-4732 (2008).

14. Fong YW, Cattoglio C, Tjian R. The intertwined roles of transcription and repair proteins. *Molecular cell* **52**, 291-302 (2013).

15. Singh I, *et al.* MiCEE is a ncRNA-protein complex that mediates epigenetic silencing and nucleolar organization. *Nature genetics* **50**, 990-1001 (2018).

16. Rubio K, *et al.* Inactivation of nuclear histone deacetylases by EP300 disrupts the MiCEE complex in idiopathic pulmonary fibrosis. *Nature communications* **10**, 2229 (2019).

17. Wang Y, Le Y, Xue JY, Zheng ZJ, Xue YM. Let-7d miRNA prevents TGF-beta1-induced EMT and renal fibrogenesis through regulation of HMGA2 expression. *Biochemical and biophysical research communications* **479**, 676-682 (2016).

18. Kugel S, *et al.* SIRT6 Suppresses Pancreatic Cancer through Control of Lin28b. *Cell* **165**, 1401-1415 (2016).
