# Supplementary material for: Positioning of nucleosomes containing γ-H2AX precedes active DNA demethylation and transcription initiation
Source: Nat Commun. 2021 Feb 16;12:1072. doi: 10.1038/s41467-021-21227-y (PMC7886895; doi:10.1038/s41467-021-21227-y)
Supplement: Supplementary file 3 — Reporting Summary [file 41467_2021_21227_MOESM3_ESM.pdf]

nature research

Corresponding author(s): Guillermo Barreto

Last updated by author(s): Dec 14, 2020

Reporting Summary

Nature Research wishes to improve the reproducibility of the work that we publish. This form provides structure for consistency and transparency in reporting. For further information on Nature Research policies, see Authors & Referees and the Editorial Policy Checklist.

Statistics

For all statistical analyses, confirm that the following items are present in the figure legend, table legend, main text, or Methods section.

n/a	Confirmed	
☐	☒	The exact sample size (*n*) for each experimental group/condition, given as a discrete number and unit of measurement
☐	☒	A statement on whether measurements were taken from distinct samples or whether the same sample was measured repeatedly
☐	☒	The statistical test(s) used AND whether they are one- or two-sided<br>*Only common tests should be described solely by name; describe more complex techniques in the Methods section.*
☐	☒	A description of all covariates tested
☐	☒	A description of any assumptions or corrections, such as tests of normality and adjustment for multiple comparisons
☐	☒	A full description of the statistical parameters including central tendency (e.g. means) or other basic estimates (e.g. regression coefficient) AND variation (e.g. standard deviation) or associated estimates of uncertainty (e.g. confidence intervals)
☐	☒	For null hypothesis testing, the test statistic (e.g. *F*, *t*, *r*) with confidence intervals, effect sizes, degrees of freedom and *P* value noted<br>*Give P values as exact values whenever suitable.*
☒	☐	For Bayesian analysis, information on the choice of priors and Markov chain Monte Carlo settings
☒	☐	For hierarchical and complex designs, identification of the appropriate level for tests and full reporting of outcomes
☒	☐	Estimates of effect sizes (e.g. Cohen's *d*, Pearson's *r*), indicating how they were calculated

Our web collection on statistics for biologists contains articles on many of the points above.

Software and code

Policy information about availability of computer code

Data collection	StepOne™ and StepOnePlus™ Software v2.3 for qRT-PCR.<br>Zeiss LSM710 confocal microscope for immunofluorescence and COMET assay data collection.<br>Infinite M200 Pro from Tecan for hydroxyproline and collagen measurements.<br>The mass data were acquired using Xcalibur x2.9-290033/2.9.0.2926 from Thermo Fisher Scientific.<br>UCSC table browser was used to download GC skew values and bed-files for mm10 annotated genes or to convert UCSC known gene names to gene symbols.
Data analysis	Excel v16.45, R-scripts and GraphPad Prism v8.2 were used to analyze the statistics. Bar graphs and box-whisker plots were made using R studio v1.2 or GraphPad Prism v8.2. Sequencing raw reads were visualized by FastQC v. 0.11.6. Low quality reads were filtered out by using trimmomatic v. 0.36. Bowtie2 v.2 2.3.4.2 or HISAT2 v.2.1.0 were used for mapping of trimmed sequencing reads. The sam files were converted to bam format by using samtools view (-Sb) followed by Samtools sort (samtools v1.3). For RNA-seq, the tag libraries were created and samples were quantified using HOMER v4.8. Differential expressed genes in IPF primary cell lines were downloaded from GEO and analysed using DEseq2 v.1.28.0. Human gene names were converted to mouse (mgi_symbol) by the use of getLDS from biomaRt program. 2D kernel density plots were generated by the help of the function kde2d from MASS package v7.3-51.4. Differential response of genes to TGFB1 was assessed by k-means clustering using Morpheus (Webtool, Broad Institute). For ChIP-seq and MNase-seq, bam files were processed and visualized using Deeptools v2.0. Further, tag libraries of ChIP-seq data were processed using HOMER v4.8 and uploaded to UCSC genome browser. pH2A.X peaks were called using MACS v.2.1.1. MaxQuant v.1.6.5.0 and MARMoSET were used to process and analyse mass spectometry data. fsgea v.1.16.0, clusterProfiler v. 3.18.0 and Webgestalt tool 2017 were used for gene ontology analysis. RNA and DNA sequences were loaded into RNA hybrid-online server and T-coffee to visualise and calculate the consensus. Bedtools intersected for ncRNAs in close proximity to the top15% genes and their localization was annotated using annotePeaks.pl from HOMER. Motif enrichment analysis was done using MEME-ChIP (MEME Suite v. 5.1.1). Geneious 8.1.9 was used for primer design and alignment of ncRNAs with gDNA.

For manuscripts utilizing custom algorithms or software that are central to the research but not yet described in published literature, software must be made available to editors/reviewers. We strongly encourage code deposition in a community repository (e.g. GitHub). See the Nature Research guidelines for submitting code & software for further information.

Data

Policy information about availability of data

All manuscripts must include a data availability statement. This statement should provide the following information, where applicable:

- Accession codes, unique identifiers, or web links for publicly available datasets
- A list of figures that have associated raw data
- A description of any restrictions on data availability

Sequencing data in MEF have been deposited in NCBI's Gene Expression Omnibus 50 and are accessible through GEO Series with accession number GSE141272. The mass spectrometry-based interactome data have been deposited into the PRIDE archive and assigned to the project accession PXD016586. Publically available ChIP-seq data from MEF and RNA-seq data from control and IPF primary fibroblasts were retrieved through the GEO Series accession number GSE63861 and GSE116086, respectively. Additionally, we used published GRO-seq in MEF (GSE76303) downloaded from GEO, DRIPc-seq files (http://rloop.bii.a-star.edu.sg/gb2_database/mm10/bw/mm9.To.mm10.GSM2104456_3T3_DRIPc_RNaseA.plus.bw) and GC skew data (http://rloop.bii.a-star.edu.sg/gb2_database/mm10/bw/mm10.gc_skew.w200.s10.bw).

Field-specific reporting

Please select the one below that is the best fit for your research. If you are not sure, read the appropriate sections before making your selection.

☒ Life sciences ☐ Behavioural & social sciences ☐ Ecological, evolutionary & environmental sciences

For a reference copy of the document with all sections, see nature.com/documents/nr-reporting-summary-flat.pdf

Life sciences study design

All studies must disclose on these points even when the disclosure is negative.

Sample size	No statistical method was used to pre-select the sample size. We performed each experiment 3 times independent from each other, which is the standard for molecular biology experiments. We consider this sufficient due to the large effect sizes observed in our experiments.
Data exclusions	No data were excluded from the analyses.
Replication	Each experiment was repeated at least 3 times, all attempts at replication were successful.
Randomization	No method of randomization was used. Randomization is not applicable to this study, as samples were not assigned to experimental groups.
Blinding	Investigators were not blinded during the experiments. In some cases, the experiments were performed by different researchers providing reproducible and statistical relevant results. Blinding was not relevant for this study, as no subjective rating (e.g. phenotypes, disease staging) of data was performed. Quantitative measurements were performed by machines.

Reporting for specific materials, systems and methods

We require information from authors about some types of materials, experimental systems and methods used in many studies. Here, indicate whether each material, system or method listed is relevant to your study. If you are not sure if a list item applies to your research, read the appropriate section before selecting a response.

Materials & experimental systems

n/a	Involved in the study	
☐	☒	Antibodies
☐	☒	Eukaryotic cell lines
☒	☐	Palaeontology
☒	☐	Animals and other organisms
☒	☐	Human research participants
☒	☐	Clinical data

Methods

n/a	Involved in the study	
☐	☒	ChIP-seq
☒	☐	Flow cytometry
☒	☐	MRI-based neuroimaging

Antibodies

Antibodies used	Reported in Material and Methods section. Primary antibodies used in this study are anti-5mC (Abcam, clone 33D3, #ab10805), anti-ACTA2 (Sigma Aldrich, #A5228), anti-AKT (Cell Signaling, #9272S), anti-COL1A1 (Sigma Aldrich, #C2456), anit-DNA-RNA hybrid (Kerafast, clone S9.6, #ENH001) anti-dsDNA (Abcam, #ab27156), anti-FN1 (Millipore, #AB2033), anti-GADD45A (Santacruz, #sc-797 sc-797, #sc-6850), anti-H3 (Abcam, #ab1791), anti-H2A.X (Millipore #07-627), anti-pH2A.X (Millipore, #05-636 clone JBW301, #07-164), anti-H4 (Abcam, #7311), anti-H2A (Abcam, #18255), anti-H2B (Millipore, #07-371), HIS-Tag (Abcam, ab9108), anti-HA-Tag (Santa Cruz #sc-805 clone Y-11), anti-HMGA2 (Abcam, #ab41878, Santacruz, #sc- 30223 and CST #8179S), anti-LMNB1 (Santacruz, #sc-6216), Mouse Control IgG (Santa Cruz, #sc-2025), Donkey-anti mouse HRP (Jackson, #715-035-150), anti-

SMAD2/3 (Cell Signaling, #3102S), anti-pSMAD2 (Cell Signaling, #3101S), Rabbit Control IgG (Santacruz, #sc-2027), anti-pPol2 (Abcam, #ab5408), anti-Pol2 (Abcam, ab26721), anti-SPT16 (Cell Signaling, #12191), anti-SSRP1 (BioLegend, 609710), clone 10D1, anti-TET1 (Active Motif, #61443), AlexaFluor488 goat-anti rabbit (Invitrogen, #A11008), AlexaFluor555 donkey-anti goat (Invitrogen, #A21432), AlexaFluor594 goat-anti mouse (Invitrogen, #A11005), ACTB (Sigma, A5316 clone AC-74) and anti-VIM (Cell Signaling, #5741S).

Validation

anti-5mC (Abcam, #ab10805) has been validated for DIP (e.g. PMID: 30954402) and was used in 103 publications.
anti-ACTA2 (Sigma Aldrich, #A5228) has been validated for Western Blotting and Immunoflurescence in human precision cut lung slices and human fibroblasts (e.g. PMID: 31110176).
anti-AKT (Cell Signaling, #9272S) validated by manufacturer using siRNA for AKT and was used in 5706 publications.
anti-COL1A1 (Sigma Aldrich, #C2456) has been validated for Western Blotting and Immunoflurescence in human precision cut lung slices and human fibroblasts (e.g. PMID: 31110176).
anit-DNA-RNA hybrid (Kerafast, clone S9.6, #ENH001) antibody has been validated in various applications (https://www.kerafast.com/productgroup/432/anti-dna-rna-hybrid-s96-antibody) and was used in 141 publications. In this study the antibody was validated by combination with an RNAseH treatment that specifically digests RNA in DNA-RNA hybrids.
anti-dsDNA (Abcam, #ab27156) has been validated by dot blot (e.g. 29289567) and was used in 47 publications.
anti-FN1 (Millipore, #AB2033) has been validated for Western Blotting and Immunoflurescence in human precision cut lung slices and human fibroblasts (e.g. PMID: 31110176).
anti-GADD45A (Santacruz, #sc-797 clone H-164, #sc-6850): #sc-797 was discontinued and replaced by #sc-6850 from the manufacturer. #sc-6850 was validated by the manufacturer by WB after overexpression of GADD45A (https://www.scbt.com/p/gadd-45alpha-antibody-c-4) and has been used in 34 publications. We have validated #sc-6850 by siRNA-mediated KD of endogenous Gadd45a.
anti-H3 (Abcam, #ab1791) was validated in various applications (https://www.abcam.com/histone-h3-antibody-nuclear-marker-and-chip-grade-ab1791.html) by the manufactuer and has been used in 3267 publications.
anti-H2A.X (Millipore #07-627) was evaluated by Western Blotting in Jurkat cell lysate by the manufacturer (https://www.emdmillipore.com/US/en/product/Anti-Histone-H2A.X-Antibody,MM_NF-07-627).
anti-pH2A.X (Millipore, #05-636 clone JBW301, #07-164) was evaluated by Western Blotting and immunohistochemestry in Jurkat cells treated with 0.5 µM staurosporine by the manufacturer (https://www.emdmillipore.com/US/en/product/Anti-phospho-Histone-H2A.X-Ser139-Antibody-clone-JBW301,MM_NF-05-636; https://www.emdmillipore.com/US/en/product/Anti-phospho-H2A.X-Ser139-Antibody,MM_NF-07-164).
anti-H2A (Abcam, #18255) was validated in various applications (https://www.abcam.com/histone-h2a-antibody-chip-grade-ab18255.html) by the manufactuer and has been used in 88 publications.
anti-H4 (Abcam, #7311) was validated for Western Blotting and ChIP (https://www.abcam.com/histone-h4-antibody-chip-grade-ab7311.html) by the manufactuer and has been used in 77 publications.
anti-H2B (Millipore, #07-371) has been validated for Western Blotting in Chicken Core Histone by the manufacturer (https://www.emdmillipore.com/US/en/product/Anti-Histone-H2B-Antibody,MM_NF-07-371).
HIS-Tag (Abcam, ab9108) was evaluated by Western Blotting in HeLa whole cell lysates transfected with a His-tagged gene (https://www.abcam.com/6x-his-tag-antibody-ab9108.html).
anti-HMGA2 (Abcam, #ab41878, Santacruz, #sc- 30223 and CST #8179S) were validated in wildtype and Hmga2-/- MEF (PMID: 26045162) by Western Blotting (#sc- 30223 and #8179S) and ChIP-seq (#ab41878).
anti-HA tag (Santa Cruz #sc-805 clone Y-11) was validated ifor Western Blotting in this study by overexpression of an HA-contstuct.
anti-LMNB1 (Santacruz, #sc-6216) has been validated by Western Blotting (e.g. PMID: 27405460).
Mouse Control IgG (Santa Cruz, #sc-2025) has been validated in human precision cut lung slices , human fibroblasts (e.g. PMID: 31110176) and mouse embryonic fibroblasts (e.g. PMID: 26045162).
Donkey-anti mouse HRP (Jackson, #715-035-150) has been validated in human precision cut lung slices , human fibroblasts (e.g. PMID: 31110176) and mouse embryonic fibroblasts (e.g. PMID: 26045162).
anti-SMAD2/3 (Cell Signaling, #3102S) was validated by Western Blotting after TGFB stimulation (e.g. PMID: 32714602).
anti-pSMAD2 (Cell Signaling, #3101S) was validated by Western Blotting after TGFB stimulation in this study.
Rabbit Control IgG (Santacruz, #sc-2027) has been validated in human precision cut lung slices , human fibroblasts (e.g. PMID: 31110176) and mouse embryonic fibroblasts (e.g. PMID: 26045162).
anti-pPol2 (Abcam, #ab5408) was validated by the manufacturer by Western Blotting and ChIP (https://www.abcam.com/rna-polymerase-ii-ctd-repeat-ysptsps-phospho-s5-antibody-4h8-chip-grade-ab5408.html). Antibody has been used in 282 studies.
anti-Pol2 (Abcam, ab26721) was validated by the manufacturer by Western Blotting and ChIP (https://www.abcam.com/rna-polymerase-ii-ctd-repeat-ysptsps-antibody-chip-grade-ab26721.html). Antibody has been used in 21 studies.
anti-SPT16 (Cell Signaling, #12191) was validated for Western Blotting and ChIP by the manufactuer (https://www.cellsignal.com/products/primary-antibodies/spt16-d7i2k-rabbit-mab/12191) and was used in 6 publications.
anti-SSRP1 (BioLegend, 609710 clone 10D1) was validated for Western Blotting and ChIP by the manufactuer (https://www.biolegend.com/en-us/products/go-chip-grade-purified-anti-ssrp1-antibody-14223) and was used in 1 publication.
anti-TET1 (Active Motif, #61443) was validated by ChIP-seq by the manufactuer (https://www.activemotif.com/catalog/details/61443/tet1-antibody-pab) and was used in 9 publications.
AlexaFluor488 goat-anti rabbit (Invitrogen, #A11008) has been validated in human precision cut lung slices and human fibroblasts (e.g. PMID: 31110176).
AlexaFluor555 donkey-anti goat (Invitrogen, #A21432) has been validated in human precision cut lung slices and human fibroblasts (e.g. PMID: 31110176).
AlexaFluor594 goat-anti mouse (Invitrogen, #A11005) was validated by the manufacturer in human IPSCs (https://www.thermofisher.com/antibody/product/Goat-anti-Mouse-IgG-H-L-Cross-Adsorbed-Secondary-Antibody-Polyclonal/A-11005) and cited in 296 publications.
anti-ACTB (Sigma, A5316 clone AC-74) has been validated in various cell lines by the manufacturer (https://www.sigmaaldrich.com/catalog/product/sigma/a5316?lang=en&region=US&cm_sp=Insite-_-caSrpResults_srpRecs_srpModel_a5316-_-srpRecs3-1). Antibody has been used in 2702 publications.
anti-VIM (Cell Signaling, #5741S) has been validated for Western Blotting and Immunoflurescence in human precision cut lung slices and human fibroblasts (e.g. PMID: 31110176).

Eukaryotic cell lines

Policy information about cell lines

Cell line source(s)	Mouse embryonic fibroblasts from timed-pregnant mice were sacrificed on post coitum day 15.5, human primary lung fibroblasts, MLE-12 (ATCC, CRL-2110) and HEK293T (ATCC, CRL-11268).
Authentication	Mouse emrbyonic fibroblasts were validated by Singh et al. 2015, Cell Research.<br>Primary human fibroblasts were obtained from UGMLC Giessen Biobank and validated by parent labs and Rubio et al.2019, Nature Communications.<br>MLE-12 (ATCC, CRL-2110) cells were validated by Singh et al. 2014, BMC Biology.<br>HEK293T (ATCC, CRL-11268) cells used for virus production were not authenticated.
Mycoplasma contamination	All cell lines used in this study were tested negative of mycoplasma contamination.
Commonly misidentified lines<br>(See ICLAC register)	No commonly misidentified lines were used.

ChIP-seq

Data deposition

☒ Confirm that both raw and final processed data have been deposited in a public database such as GEO.

☒ Confirm that you have deposited or provided access to graph files (e.g. BED files) for the called peaks.

Data access links<br>*May remain private before publication.*	Sequencing data of ChIP samples have been deposited in NCBI's Gene Expression Omnibus and are accessible through GEO Series with accession number GSE141272.
Files in database submission	ChIP_WT_Input_001_R1.fastq<br>ChIP_WT_Input_002_R1.fastq<br>ChIP_WT_Input_003_R1.fastq<br>ChIP_WT_H2AX_001_R1.fastq<br>ChIP_WT_H2AX_002_R1.fastq<br>ChIP_WT_H2AX_003_R1.fastq<br>ChIP_WT_H3_001_R1.fastq<br>ChIP_WT_H3_002_R1.fastq<br>ChIP_WT_H3_003_R1.fastq<br>ChIP_WT_H3_004_R1.fastq<br>ChIP_WT_pPolII_001_R1.fastq<br>ChIP_WT_pPolII_002_R1.fastq<br>ChIP_WT_pPolII_003_R1.fastq<br>ChIP_WT_Gadd45a_001_R1.fastq<br>ChIP_WT_Gadd45a_002_R1.fastq<br>ChIP_WT_Gadd45a_003_R1.fastq<br>ChIP_KO_Input_001_R1.fastq<br>ChIP_KO_Input_002_R1.fastq<br>ChIP_KO_Input_003_R1.fastq<br>ChIP_KO_H2AX_001_R1.fastq<br>ChIP_KO_H2AX_002_R1.fastq<br>ChIP_KO_H2AX_003_R1.fastq<br>ChIP_KO_H3_001_R1.fastq<br>ChIP_KO_H3_002_R1.fastq<br>ChIP_KO_H3_003_R1.fastq<br>ChIP_KO_H3_004_R1.fastq<br>ChIP_KO_pPolII_001_R1.fastq<br>ChIP_KO_pPolII_002_R1.fastq<br>ChIP_KO_pPolII_003_R1.fastq<br>ChIP_KO_Gadd45a_001_R1.fastq<br>ChIP_KO_Gadd45a_002_R1.fastq<br>ChIP_KO_Gadd45a_003_R1.fastq<br>ChIP_KO_Gadd45a_004_R1.fastq<br>ChIP_WT_Ctrl_Input_R1.fastq<br>ChIP_WT_TGFB1_Input_R1.fastq<br>ChIP_KO_Ctrl_Input_R1.fastq<br>ChIP_KO_TGFB1_Input_R1.fastq<br>ChIP_WT_Ctrl_pH2AX_R1.fastq<br>ChIP_WT_TGFB1_pH2AX_R1.fastq<br>ChIP_KO_Ctrl_pH2AX_R1.fastq<br>ChIP_KO_TGFB1_pH2AX_R1.fastq<br>ChIP_WT_Ctrl_SUPT16H_R1.fastq<br>ChIP_KO_Ctrl_SUPT16H_R1.fastq

ChIP_WT_Ctrl_SSRP1_R1.fastq
ChIP_KO_Ctrl_SSRP1_R1.fastq

Genome browser session (e.g. UCSC)	NA

Methodology

Replicates	One sample was sent for sequencing experiements.
Sequencing depth	Quality measures can for ChIP-seq can be found in Figure S1A, Figure S2E and S6C.
Antibodies	Reported in Material and Methods section. Primary antibodies used in this study are anti-H2A.X (Millipore #07-627), anti-pH2A.X (Millipore, #05-636), anti-GADD45A (Santacruz, #sc-797), anti-H3 (Abcam, #ab1791), anti-pPol2 (Abcam, #ab5408), anti-SPT16 (Cell Signaling, #12191) and anti-SSRP1 (BioLegend, 609710).
Peak calling parameters	Peak calling was performed by using model-based analysis for ChIP-seq (MACS) with a cut-off of p<0.01.
Data quality	Raw reads were visualized by FastQC to determine the quality of the sequencing. Low quality reads were removed using trimmomatic v0.32. Peak calling was performed by using model-based analysis for ChIP-seq (MACS) with a cut-off of p<0.01.
Software	Sequencing raw reads were visualized by FastQC. Low quality reads were filtered out by using trimmomatic. Bowtie2 was used for mapping of trimmed sequencing reads. The sam files were converted to bam format by using samtools view (-Sb) followed by Samtools sort. Bam files were processed and visualized using Deeptools. Further, tag libraries of ChIP-seq data were processed using HOMER and uploaded to UCSC genome browser. pH2A.X peaks were called using MACS.
